# Network Analysis of Symptom Structures in Autism, Schizophrenia, and Non-Diagnosed Controls

**DOI:** 10.1192/j.eurpsy.2025.751

**Published:** 2025-08-26

**Authors:** S. Holka, D. Sörnyei, Á. Vass, K. Farkas

**Affiliations:** 1 Department of Psychiatry and Psychotherapy, Semmelweis University; 2 HUN-REN Institute of Cognitive Neuroscience and Psychology, Research Centre of Natural Sciences; 3 Department of Clinical Psychology, Semmelweis University, Budapest, Hungary

## Abstract

**Introduction:**

Despite differences in the onset and symptomatology, Autism Spectrum Disorder (ASD) and Schizophrenia (SCH) are neurodevelopmental conditions with evolving conceptual links in modern psychiatry. This stems from increasing evidence suggesting overlap in social cognition, attachment and the conceptualization as disorders of the self. A transdiagnostic approach, in which network analysis can play an important role, offers valuable insights into the complex interrelationships between symptoms and key constructs in the psychopathology of them. It may reveal underlying similarities and differences, contributing to more targeted interventions.

**Objectives:**

Our study aims to investigate the symptom structures of ASD, SCH, and neurotypical individuals (NTP) using network analysis. By comparing them, our explorative goal was to identify key constructs and their connections, providing potential intervention targets. We hypothesize that both ASD and SCH networks will significantly differ from the NTP network, and that mentalization and disorganized schizotypy would be the most central nodes in the networks of ASD and SCH.

**Methods:**

In a cross-sectional study, 1694 participants were involved in the analysis (NNTP=1477, NASD=155, NSCH=62). Participants completed self-report questionnaires. Based on theoretical and methodological considerations we included psychological inflexibility, mentalization, insecure attachment, perceived social support, minimal and narrative self, negative and disorganized schizotypy, autistic traits, anxiety in the analysis. Gaussian Graphical Models were used to estimate relationships between constructs, with LASSO regularization, focusing on network centrality and predictability measures. Network Comparison Test was applied to unveil local and global differences.

**Results:**

A comparative representation with node scaling for predictability values are shown in image 1 and image 2. Minimal self was the most predictable node in each case. Central nodes in the ASD network were psychological inflexibility and minimal self, in the SCH network narrative self and insecure attachment, in the NTP network psychological inflexibility and minimal self. Significant differences in global strength were observed between ASD and NTP networks. Details of a relevant pattern are shown on image 3.

**Image 1:**

**Figure:**
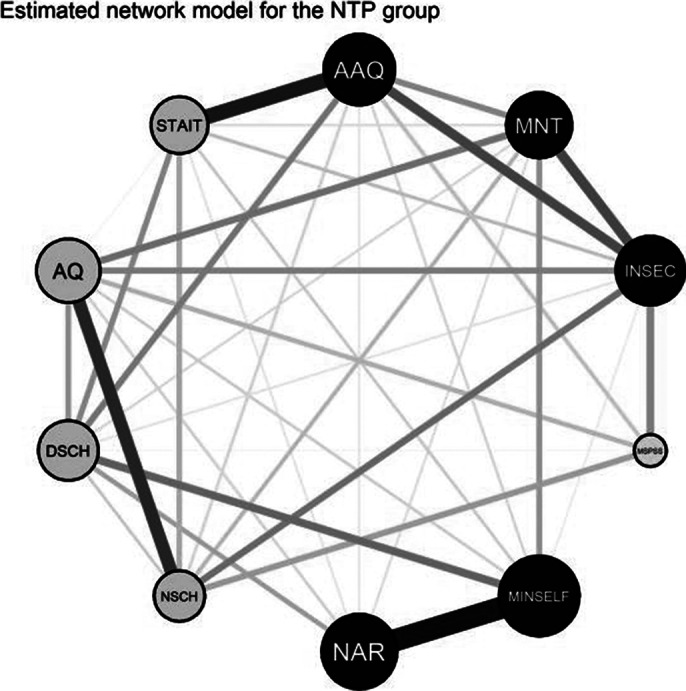

**Image 2:**

**Figure:**
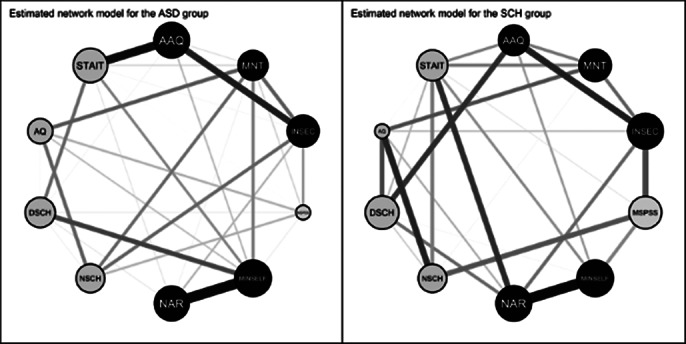

**Image 3:**

**Figure:**
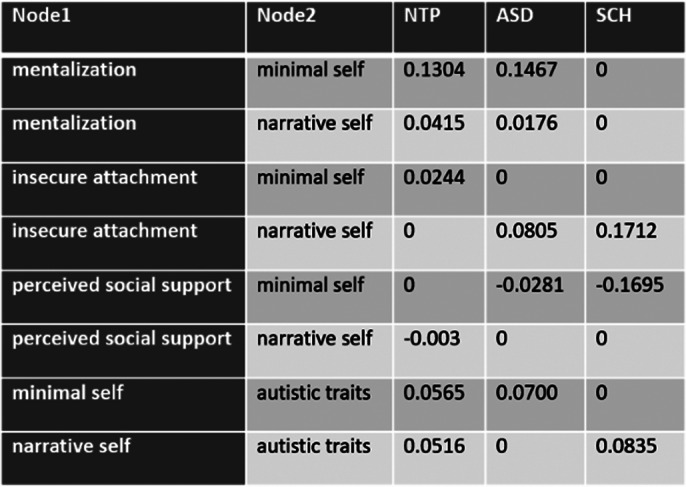

**Conclusions:**

Our study highlights distinct symptom networks in ASD and SCH, with distinct centralities emerging. Results suggest interventions targeting psychological inflexibility and self-concept may be effective for ASD, while in SCH, narrative self experience and attachment insecurity may be beneficial. Results show that focusing on isolated constructs may overlook the importance of other constructs. By focusing to the strongest edges and relevant patterns, clinicians may benefit from interventions that simultaneously target the dimensions of the relationships, also considering the most central nodes in the network.

**Disclosure of Interest:**

None Declared

